# SNP and SCAR Markers for Specific Discrimination of Antler-Shaped *Ganoderma lucidum*

**DOI:** 10.3390/microorganisms7010012

**Published:** 2019-01-09

**Authors:** O-Chul Kwon, Chang-Soo Lee, Young-Jin Park

**Affiliations:** 1 Sericultural & Apicultural Materials Division, National Academy of Agricultural Science, Rural Development Administration, Wanju-gun 565-851, Korea; jiny5462@daum.net; 2 Department of Integrated Biosciences, Research Institute for Biomedical & Health Science, College of Biomedical and Health Science, Konkuk University, Chungju-si 27478, Korea; cslee@kku.ac.kr

**Keywords:** antler-shape, *Ganoderma lucidum*, kidney-shape, mitochondrial SSU rDNA, SCAR marker, SNP marker

## Abstract

In this study we identified single nucleotide polymorphism (SNP) and sequence characteristic amplification region (SCAR) markers for specific identification of antler-shaped *Ganoderma lucidum* strains. When the partial mitochondrial SSU rDNA gene sequence of various antler- and kidney-shaped *G. lucidum* strains were analyzed and aligned, an SNP was found only in the antler-shaped *G. lucidum* strain at position 456 bp. In addition, this SNP of antler-shaped strains was digested by *HinfI* restriction enzyme. We further analyzed the polymorphism of various *G. lucidum* strains by random amplified polymorphic DNA (RAPD) analysis. In RAPD analysis, we isolated and sequenced a fragment, specific for antler-shaped *G. lucidum* strains. Based on this specific fragment sequence, two sets of specific primer pairs for antler-shaped *G. lucidum* strains were designed. PCR analysis revealed that two specific bands were observed only from antler-shaped strains. These two molecular markers will be helpful for identification of morphological characteristics of *G. lucidum*.

## 1. Introduction

*Ganoderma lucidum* (Curtis) P. Karst. was named by Petter Adolf Karsten in 1881 based on material from England [1,2]. The name *G. lucidum* has been applied to collections from various countries, including East Africa, Oceania, North America, South America, Asia (China, Japan, and Korea), and Europe [3]. *G. lucidum* has been used in traditional medicine for thousands of years in East Asian countries, such as China, Japan, and Korea. The bioactive compounds such as flavonoids, ganoderic acid, phenolics, and polysaccharides of *G. lucidum* have been reported to show immunomodulatory effect, antitumor activity, and inhibitory activity against histamine release and cholesterol synthesis [4,5,6,7]. Antler-shaped *G. lucidum* is a variant of *G. lucidum* that is rarely found in nature [8]. This rare variant is famous for its medicinal effect in China and Japan [9]. It is commonly called “Lu jiao Lingzhi” in China, “Rokkaku-Reishi” in Japan [10], and “Nokgak Yeongji” in Korea. Antler-shaped *G. lucidum* has been reported to contains much larger amounts of β-D-glucans and triterpenoids than kidney-shaped *G. lucidum* [11,12]. It is also known to be more effective in immunostimulatory and anti-tumor activities than kidney-shaped *G. lucidum* [12,13]. Therefore, antler-shaped *G. lucidum* is expected to show much stronger pharmacological activity. On phylogenetic analyses of the genus, *G. lucidum* from different parts of the world were reported to belong to several separated lineages [14,15,16,17,18]. Regarding research on the markers for *Ganoderma* species, there are several reports about taxonomic diversity by RAPD, RFLP, AFLP, and other methods [19,20,21,22]. However, there is no marker for specific identification of antler-shaped *G. lucidum* strain.

Molecular markers are developed by several techniques, such as amplified fragment length polymorphism (AFLP), polymerase chain reaction-restriction fragment length polymorphism (PCR-RFLP), random amplified polymorphic DNA (RAPD), and sequence characterized amplification region (SCAR) [23]. Among these techniques, PCR-RFLP and RAPD are the quickest and relatively simplest methods and are particularly useful for genetic variation and mutation analyses [19,24]. Single nucleotide polymorphisms (SNPs) have an important role in biomedical and biological researches, including genetic variations, mutations, and investigation of complex genetic diseases [24,25]. SNPs are found most commonly between DNA sequences obtained from different individuals or the same individuals [26,27,28,29]. Therefore, SNP is the most abundant marker system in animal, plant, and microorganism genomes and has recently emerged as the new generation molecular marker for various applications. Furthermore, SNP based technique allows the successful detection and distinction of specific genetic variations even in a low diversity species [30]. The target SNPs are distinguished by digestion using specific restriction enzymes in a process called “PCR-RFLP” [24]. In addition, the combination of RAPD and SCAR markers is a simple and useful tool for molecular analysis or genetic characterization of different species [31].

*Ganoderma* species has different morphological characteristics due to environmental factors and genetic variations [32]. In addition, development of cultivation techniques has enabled the formation of antler-shaped pileus in China, Japan, and Korea [8,13]. Thus, genetic characterization and accurate identification of antler-shaped *G. lucidum* are important. In this study, we attempted to develop SNP and SCAR markers for specific identification of antler-shaped *G. lucidum* at the mycelial stage. These SNP and SCAR markers will be helpful in genetically identifying the morphological characteristics of *G. lucidum*.

## 2. Materials and Methods

### 2.1. Strains and Culture Conditions

Five antler- and nineteen kidney-shaped *G. lucidum* strains were collected from the Mushroom Division of the Rural Development Administration (Eumseong, Korea), Incheon University (Incheon, Korea), the Korean Agricultural Culture Collection (KACC, Suwon, Korea), the Korean Collection for Type Culture (KTCT, Jeongeup, Korea) (Table 1). In addition, commercial kidney-shaped *G. lucidum* (Imsil, Korea) was purchased and used in this study. *G. lucidum* mycelia were cultured in potato dextrose broth (PDB; Difco, Detroit, MI, USA) at 28 °C for 2 weeks.

### 2.2. DNA Extraction and Amplification

Cultured mycelia [filtered through 2 layers of MiraCloth (Calbiochem, La Jolla, CA, USA)] and fruiting bodies were ground in liquid nitrogen, and genomic DNA was extracted using the cetyltrimethylammonium bromide (CTAB) method [33]. Samples (0.5 g) were mixed with 400 mL of extraction buffer (100 mM NaCl, 50 mM EDTA, 0.25 M Tris-HCl, 5% SDS) and 400 mL of 2 × CTAB buffer (2% CTAB, 100 mM Tris-Hcl pH 8.0, 20 mM EDTA pH 8.0, 1.4 M NaCl, 1% polyvinyl pyrrolidone). The extracted DNA was clarified with an extraction solution (phenol–chloroform–isoamyl alcohol, 25:24:1) and then was precipitated with 1/30 volume of 3 M sodium acetate and 1 volume of isopropanol. Purified DNA was sequentially washed with 70% ethanol and dried. The DNA pellet was dissolved in 60 μL of TE buffer (10 mM Tris-HCl, 1 mM EDTA, pH 8.0) and treated sequentially with 6 μL of RNase A (20 mg/mL).

The extracted DNA was used as a template (adjusted to 100 ng/µL) for PCR amplification of the partial mitochondrial SSU rDNA gene and for RAPD analysis. All PCR reactions were performed with a premixed polymerase kit (Taq PreMix; TNT Research, Anyang, Korea) in a 20 μL reaction mixture containing 1 μL DNA (100 ng/μL) and 2.5 pmol of each primer. All PCR primer sequences used are shown in Table 2. Partial mitochondrial SSU rDNA gene was amplified using primer pairs of BMS 105 and BMS 173 [34]. Amplification conditions for the mitochondrial SSU rDNA were 3 min of initial denaturation at 94 °C, followed by 25 cycles of denaturation at 94 °C for 30 s, annealing at 55 °C for 30 s, and extension at 72 °C for 2 min, and final extension at 72 °C for 10 min using TaKaRa Thermal cycler (TaKaRa, Tokyo, Japan). RAPD-PCR amplification conditions were 10 min of initial denaturation at 94 °C, followed by 35 cycles of denaturation at 94 °C for 1 min, annealing at 55 °C for 1 min, and extension at 72 °C for 2 min, and final extension at 72 °C for 7 min using TaKaRa Thermal cycler (TaKaRa, Tokyo, Japan). PCR products were detected by electrophoresis on 1.2% agarose gel in 0.5 × TAE buffer (Tris-acetic acid-EDTA), stained with ethidium bromide (EtBr), and visualized on a UV transilluminator. The PCR product sizes were determined by comparison to 1 kb Plus Ladder Marker (TNT research, Anyang, Korea).

### 2.3. Cloning, Sequencing and Sequence Analysis

The PCR products and specific DNA fragments were ligated into pGEM-T easy vector (Promega, Madison, WI, USA), according to the manufacturer’s instruction. After ligation, the plasmids were transformed into competent cell (*E. coli* DH5α; RBC, New Taipei City, Taiwan) by the heat-shock method [35]. Plasmid DNAs were extracted using FavorPrep™ Plasmid Extraction Kit (Favorgen Biotech Corporation, Pingtung, Taiwan). Insert DNAs of the recombinant plasmids were confirmed by restriction enzyme *EcoRI*. Sequences were determined by a commercial service (Genotech, Daejeon, Korea) and analyzed using the BioEdit program (http://www.mbio.ncsu.edu/bioedit/ bioedit.html).

### 2.4. SNP Detection and Validation

Restriction enzyme capable of cleaving antler-shaped *G. lucidum* specific SNP within partial mitochondrial SSU rDNA sequences was analyzed using SeqBuilder program (DNAStar, Inc., Madison, Wis., USA). PCR products of partial mitochondrial SSU rDNA gene were digested by the restriction enzyme FastDigest *HinfI* (Fermentas, Vilnius, Lithuania). The restriction enzyme digestion reaction was done by mixing 5 μL of PCR products in 0.5 μL of restriction enzyme and 1.2 μL of 10 × FastDigest buffer. The total volume was made up to 12 μL using autoclaved distilled water and was then incubated for 10 min at 37 °C. The digested product was visualized by electrophoresis in 1.2% agarose gel using 1 kb Plus Ladder Marker (TNT research, Anyang, Korea).

### 2.5. SCAR Primer Design and Validation

Specific DNA fragments were eluted using the Qiaquick Gel Extraction Kit (Qiagen INC., Chatsworth, CA, USA), according to the manufacturer’s instruction. Two sets of specific primer pairs for antler-shaped *G. lucidum* were designed for SCAR marker. PrimerSelect in Lasergene (DNAStar, Inc. Madison, WI, USA) was used for the primer design. Specific primers are shown in Table 2. PCR reactions of specific primer pairs for antler-shaped *G. lucidum* were performed in a total volume of 20 μL, containing 1 μL of DNA (100 ng/μL) and 2.5 pmol of each primers (KAGL 1F/1R and KAGL 2F/2R primer pairs). The PCR conditions were 5 min of initial denaturation 94 °C, followed by 27 cycles of denaturation at 94 °C for 15 s, annealing at 61 °C for 15 s, and extension at 72 °C for 30 s, and final extension at 72 °C for 10 min using TaKaRa Thermal cycler (TaKaRa, Tokyo, Japan). The PCR product was visualized by electrophoresis in 1.2% agarose gel using 1 kb Plus Radder Marker (TNT research, Anyang, Korea).

### 2.6. Cultivation of G. lucidum Fruit Body

Sawdust mixed with rice bran in 4:1 ratio was watered and placed into polypropylene bottles. The substrate was sterilized at 121 °C for 40 min in an autoclave and cooled at room temperature for 24 h. Then, the sawdust medium was inoculated with the cultured *Ganoderma* mycelium in potato dextrose agar (PDA; Difco, Detroit, MI, USA) medium. The inoculated sawdust media were incubated at 28 °C for approximately one month until mycelia spread all over the media. When the mycelium had colonized the substrate completely, it was transferred to a fruiting room at 26 °C. The substrate was wetted to increase the moisture content to approximately 60%–80%. The artificially induced formation of antler shape from kidney shape was achieved under dark and 0.1% CO_2_ conditions.

## 3. Results

### 3.1. SNP Analysis of Partial Mitochondrial SSU rDNA Gene

**Figure 1:**
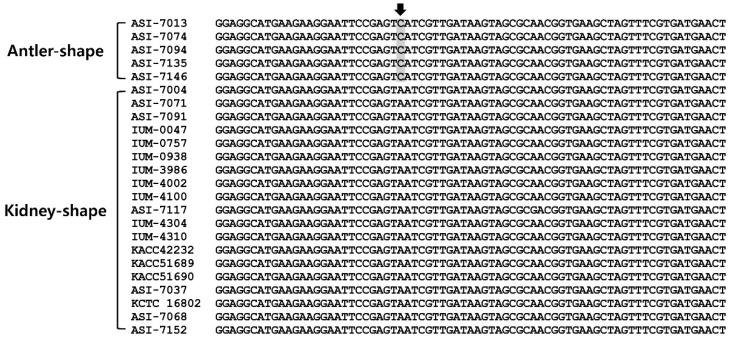
Alignment of partial mitochondrial SSU rDNA sequences of *Ganoderma lucidum* strains. Arrow indicates the single nucleotide polymorphism (SNP) found from antler-shaped *G. lucidum* strains.

**Figure 2:**
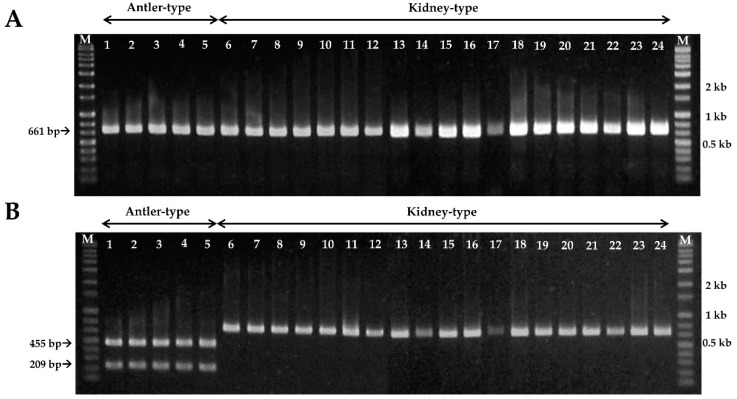
PCR-RFLP of antler- and kidney-shaped *Ganoderma lucidum*. (**A**) PCR amplification products in partial mitochondrial SSU rDNA gene containing the SNP location from *G. lucidum*; (**B**) The result of digestion with *HinfI* restriction enzyme from the PCR products. The arrow indicates the restriction fragment sizes. Lanes 1–5: antler–shaped *G. lucidum*. Lanes 6–24: kidney-shaped *G. lucidum* (numbers 1–24, respectively, in Table 1). M: size markers (1 kb ladder).

In PCR analysis, the amplified PCR product sizes from the partial mitochondrial SSU rDNA gene sequences of the antler- and kidney-shaped *G. lucidum* were of identical length of 661-bp (Figure 1, Figure 2A and Figure S1). Among them, SNPs were found only in the antler-shaped *G. lucidum* strains at location 456 bp (Figure 1). At 456 bp location, antler-shaped *G. lucidum* strains contain the nucleotide cytosine (C) but kidney-shaped counterparts contain the nucleotide adenine (A). This SNP region in antler-shaped *G. lucidum* is recognized by the *HinfI* restriction enzyme. Consequently, the PCR products of the antler-shaped *G. lucidum* strains were digested to 209-bp and 455-bp sizes by *HinfI* restriction enzyme. (Figure 2B). Thus, antler- and kidney-shaped *G. lucidum* strains could be distinguished by *HinfI* restriction enzyme.

### 3.2. Development of SCAR Marker for Antler-Shaped G. lucidum

**Figure 3:**
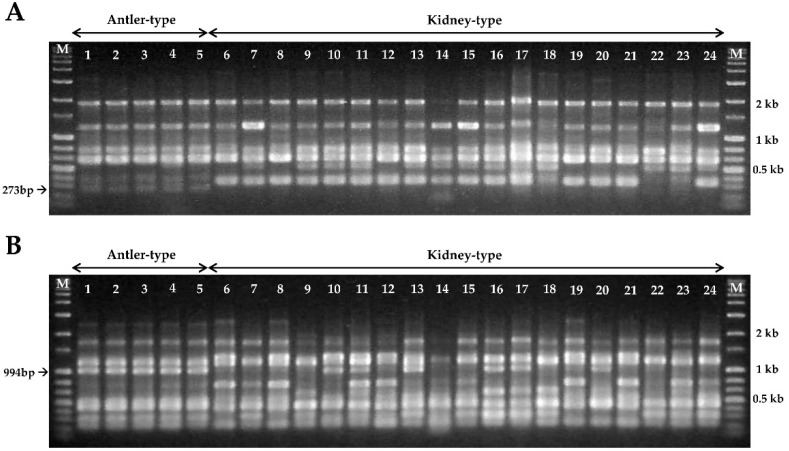
RAPD analysis of 24 *Ganoderma lucidum* strains using the (**A**) URP 1 and (**B**) URP 5 primers. The arrows indicate the specific fragment sizes of antler-shaped *G. lucidum* strains. Lanes 1–5: antler-shaped *G. lucidum*. Lanes 6–24: kidney-shaped *G. lucidum* (numbers 1–24, respectively, in Table 1). M: size markers (1 kb ladder).

In this study, 12 URP primers were used to evaluate the specific polymorphism of *G. lucidum* strains. Among them, the URP1 and URP5 primers revealed a good polymorphic amplification pattern for antler- and kidney-shaped *G. lucidum* strains. In addition, amplification with URP1 and URP5 primers from all antler-shaped *G. lucidum* strains showed specific DNA bands of 273-bp and 994-bp, respectively (Figure 3). The two target DNA bands (273-bp and 994-bp) for the specific identification of antler-shaped *G. lucidum* were isolated and sequenced to design strain-specific primers.

**Figure 4:**
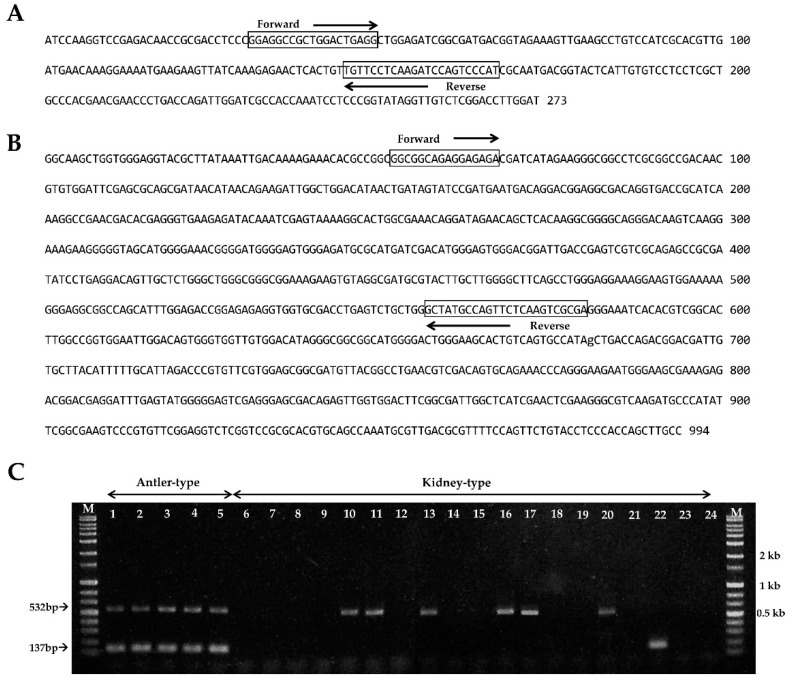
Primers for specific detection of antler-shaped *Ganoderma lucidum* and PCR amplification. The specific DNA fragments sequences amplified with (**A**) URP 1 and (**B**) URP 5 from the antler-shaped *G. lucidum*. Black boxes indicate the primer positions; (**C**) PCR amplification. Arrows indicate the antler-shaped *G. lucidum*-specific amplified fragments. Lanes 1–5: antler-shaped *G. lucidum*. Lanes 6–24: kidney-shaped *G. lucidum* (numbers 1–24, respectively, in Table 1). M: size markers (1 kb ladder).

The DNA fragment sequences and specific primers of antler-shaped *G. lucidum* strain are shown in Figure 4A,B. As predicted, the PCR results from all of antler-shaped *G. lucidum* strains were found to have two specific DNA bands in 137-bp and 532-bp (Figure 4C). However, either one of the two specific bands (137-bp or 532-bp) or no band was observed from kidney-shaped *G. lucidum* strains (Figure 4C). This result indicates that two sets of specific primer pairs (KAGL 1F/1R and KAGL 2F/1R) are specific to antler-shaped *G. lucidum* and could be used to differentiate it from kidney-shaped *G. lucidum*.

**Figure 5:**
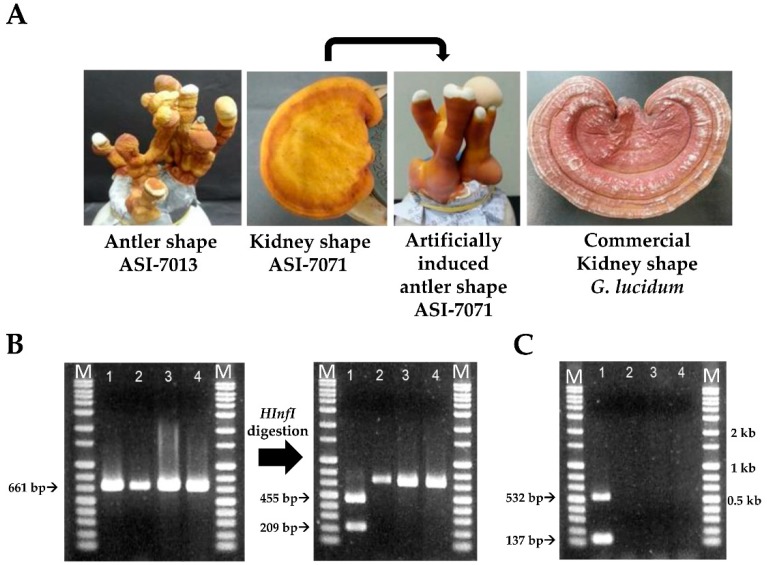
SNP and SCAR markers for specific identification of antler-shaped *Ganoderma lucidum* fruit body. (**A**) Morphology of antler- and kidney-shaped *G. lucidum*; (**B**) SNP marker validation for antler-shaped fruit bodies by *HinfI* digestion; (**C**) SCAR marker validation for antler-shaped fruit bodies. Lane 1: antler-shaped *G. lucidum* (ASI-7013). Lane 2: Kidney-shaped *G. lucidum* (ASI-7071). Lane 3: artificially induced antler-shape (ASI-7071). Lane 4: commercial *G. lucidum* of kidney-shape. M: size markers (1 kb ladder).

We checked SNP and SCAR markers for the specific identification of antler-shaped *G. lucidum* fruit body and artificial forming of antler shape (Figure 5). Consequently, SNP marker was confirmed to have two fragments of the sizes 209-bp and 455-bp by *HinfI* restriction enzyme digestion in the antler-shaped *G. lucidum* fruit body (ASI-7013), except for kidney-shaped *G. lucidum* fruit bodies (ASI-7071 and commercial *G. lucidum*) and artificial forming antler-shape from kidney-shape (ASI-7071) (Figure 5B). Furthermore, SCAR marker revealed that both of two specific bands were found only in the fruit body of antler-shaped *G. lucidum* strain (ASI-7013) (Figure 5C). These results showed that SNP and SCAR markers can help distinguish between the antler- or kidney-shaped *G. lucidum* fruit body.

## 4. Discussion

This study aimed to develop the SNP and SCAR markers for specific identification of antler-shaped *Ganoderma lucidum* strains. *G. lucidum* has been widely used as a valuable medicinal agent because of its wide variety of anti-inflammatory, antitumor, antioxidant, and other biological activities [36,37]. It has been reported that antler-type *G. lucidum* produces higher levels of bioactive compounds such as flavonoids, ganoderic acid, and phenolics than kidney-shaped *G. lucidum* [11,12,38].

Mitochondrial DNA is one of the most important genetic resources and it is used as a marker for various phylogenetic classifications [39]. In addition, Hong et al. [34] reported that the information of valuable domains in mitochondrial SSU rDNA gene was useful in phylogenetic analysis of the *Ganoderma* species. In this study, we analyzed the partial mitochondrial small-subunit ribosomal DNA gene sequence of various antler- and kidney-shaped *G. lucidum* strains. We found that the antler-shaped *G. lucidum* has an SNP in the mitochondrial SSU rDNA gene sequence and can be digested by the *HinfI* restriction enzyme.

We also designed two sets of specific primer pairs to develop SCAR-marker for the antler-shaped *G. lucidum* strains based on RAPD analysis. PCR analysis with antler-shaped specific-primers revealed that the artificially induced antler-shaped *G. lucidum* could also be specifically discriminated. RAPD with random arbitrary primers has been widely used in genetic diversity studies of fungi [40,41,42]. In addition, genetic relationships can also be inferred even within fungal pathogen species using RAPD markers. Manulis et al. [43] reported that specific banding patterns from RAPD were subsequently used as probes to distinguish between races of the carnation wilt fungal pathogen *Fusarium oxysporum* f. sp. *dianthi*. Moreover, RAPD markers have been reported to be useful in diagnostic studies of fungal pathogens such as *Alternaria* species (the causal agent of brown spot of citrus) and *Leptosphaeria maculans* (the causal agent of blackleg of crucifers) [40,44]. However, RAPD analysis using random arbitrary primers tends to result in low reproducibility. Universal rice primers (URPs) developed from the repetitive sequences of rice genome can be used for PCR fingerprinting of various organisms including plants, animals, and microorganisms due to their high reproducibility [45]. In addition, RAPD analysis using URP primers is a useful tool for the characterization and grouping of fungal species at intraspecific and interspecific levels [46,47,48,49,50].

Antler-shaped *G. lucidum* is a valuable herbal medicine in China, Japan, and Korea. Antler-shaped *G. lucidum* can be cultivated naturally or artificially. A dark condition with poor ventilation does not expand the pileus. In addition, high levels of carbon dioxide (CO_2_) have been reported to support the production of antler-shaped fruiting bodies [9,38]. Thus, its morphology can be changed by artificially modulating the cultivation conditions. Antler-shaped *G. lucidum* strain has been mainly distinguished by morphological characteristics. However, this form is not easy to distinguish because of the various formulations and development of cultivation techniques. Therefore, the SNP and SCAR markers for the identification of antler-shaped *G. lucidum* strains will be useful for protection of the breed, breeding, time saving, and the cost-effective part.

## Figures and Tables

**Table 1:** *Ganoderma lucidum* strains used in this study.

No.	Species	Collection	Origin	Shape
1	*Ganoderma lucidum*	^1^ ASI-7013	Korea	antler
2	*Ganoderma lucidum*	ASI-7135	Korea	antler
3	*Ganoderma lucidum*	ASI-7146	Korea	antler
4	*Ganoderma lucidum*	ASI-7074	Korea	antler
5	*Ganoderma lucidum*	ASI-7094	Korea	antler
6	*Ganoderma lucidum*	ASI-7004	Korea	kidney
7	*Ganoderma lucidum*	ASI-7071	Korea	kidney
8	*Ganoderma lucidum*	ASI-7091	Korea	kidney
9	*Ganoderma lucidum*	ASI-7117	Korea	kidney
10	*Ganoderma lucidum*	^2^ IUM-0047	Korea	kidney
11	*Ganoderma lucidum*	IUM-0757	Korea	kidney
12	*Ganoderma lucidum*	IUM-0938	Korea	kidney
13	*Ganoderma lucidum*	IUM-3986	Korea	kidney
14	*Ganoderma lucidum*	IUM-4002	Korea	kidney
15	*Ganoderma lucidum*	IUM-4100	Korea	kidney
16	*Ganoderma lucidum*	IUM-4304	Bangladesh	kidney
17	*Ganoderma lucidum*	IUM-4310	Bangladesh	kidney
18	*Ganoderma lucidum*	^3^ KACC42232	Japan	kidney
19	*Ganoderma lucidum*	KACC51689	Japan	kidney
20	*Ganoderma lucidum*	KACC51690	Japan	kidney
21	*Ganoderma lucidum*	ASI-7037	Papuanewguinea	kidney
22	*Ganoderma lucidum*	^4^ KCTC 16802	Thailand	kidney
23	*Ganoderma lucidum*	ASI-7068	USA	kidney
24	*Ganoderma lucidum*	ASI-7152	Korea	kidney
25	*Ganoderma lucidum*	Commercial strain	Korea	Kideny

^1^ Agricultural Science Institute, ^2^ Incheon University Mushroom, ^3^ Korean Agricultural Culture Collection, ^4^ Korean Collection for Type Cultures.

**Table 2:** Primers used in this study.

Primer	Sequences (5′—3′)	Target
BSM105_F	ATTAGTCGGTCTCGAAGCAAACG	Partial mitochondrial SSU rDNA gene
BSM173_R	TGCTATGACTTTTGAGATGTTAC	Partial mitochondrial SSU rDNA gene
URP 1	ATCCAAGGTCCGAGACAACC	^1^ RAPD
URP 5	GGCAAGCTGGTGGGAGGTAC	RAPD
KAGL1_F	GGAGGCCGCTGGACTGAGG	Antler-specific
KAGL1_R	ATGGGACTGGATCTTGAGGAACA	Antler-specific
KAGL2_F	GGCGGCGGCAGAGGAGAG	Antler-specific
KAGL2_R	TCGCGACTTGAGAACTGGCATAGC	Antler-specific

^1^ Random Amplified Polymorphic DNA.

## Supplementary Materials

The following figures are available online at http://www.mdpi.com/2076-2607/7/1/12/s1. Figure S1: Alignment of partial mitochondrial SSU rDNA sequences of *Ganoderma lucidum* strains.
