# Supplementary material for: SNP and SCAR Markers for Specific Discrimination of Antler-Shaped Ganoderma lucidum
Source: Microorganisms. 2019 Jan 9;7(1):12. doi: 10.3390/microorganisms7010012 (PMC6352128; doi:10.3390/microorganisms7010012)
Supplement: Supplementary file 1 [file microorganisms-07-00012-s001.pdf]

```
                   10        20        30        40        50        60        70        80        90       100       110       120
          ....|....|....|....|....|....|....|....|....|....|....|....|....|....|....|....|....|....|....|....|....|....|....|....|
ASI-7013   ATTAGTCGGTCTCGAAGCAAACGGAGTGAAGCATGTTATTTAATACGATAATCCGCGTAAAACCTTACCAGAACTTGCATACAAACTATAAAATTTCCTACCTGAAAAGGTAAAGAAAAT
ASI-7074   ATTAGTCGGTCTCGAAGCAAACGGAGTGAAGCATGTTATTTAATACGATAATCCGCGTAAAACCTTACCAGAACTTGCATACAAACTATAAAATTTCCTACCTGAAAAGGTAAAGAAAAT
ASI-7094   ATTAGTCGGTCTCGAAGCAAACGGAGTGAAGCATGTTATTTAATACGATAATCCGCGTAAAACCTTACCAGAACTTGCATACAAACTATAAAATTTCCTACCTGAAAAGGTAAAGAAAAT
ASI-7135   ATTAGTCGGTCTCGAAGCAAACGGAGTGAAGCATGTTATTTAATACGATAATCCGCGTAAAACCTTACCAGAACTTGCATACAAACTATAAAATTTCCTACCTGAAAAGGTAAAGAAAAT
ASI-7146   ATTAGTCGGTCTCGAAGCAAACGGAGTGAAGCATGTTATTTAATACGATAATCCGCGTAAAACCTTACCAGAACTTGCATACAAACTATAAAATTTCCTACCTGAAAAGGTAAAGAAAAT
ASI-7004   ATTAGTCGGTCTCGAAGCAAACGGAGTGAAGCATGTTATTTAATACGATAATCCGCGTAAAACCTTACCAGAACTTGCATACAAACTATAAAATTTCCTACCTGAAAAGGTAAAGAAAAT
ASI-7071   ATTAGTCGGTCTCGAAGCAAACGGAGTGAAGCATGTTATTTAATACGATAATCCGCGTAAAACCTTACCAGAACTTGCATACAAACTATAAAATTTCCTACCTGAAAAGGTAAAGAAAAT
ASI-7091   ATTAGTCGGTCTCGAAGCAAACGGAGTGAAGCATGTTATTTAATACGATAATCCGCGTAAAACCTTACCAGAACTTGCATACAAACTATAAAATTTCCTACCTGAAAAGGTAAAGAAAAT
IUM-0047   ATTAGTCGGTCTCGAAGCAAACGGAGTGAAGCATGTTATTTAATACGATAATCCGCGTAAAACCTTACCAGAACTTGCATACAAACTATAAAATTTCCTACCTGAAAAGGTAAAGAAAAT
IUM-0757   ATTAGTCGGTCTCGAAGCAAACGGAGTGAAGCATGTTATTTAATACGATAATCCGCGTAAAACCTTACCAGAACTTGCATACAAACTATAAAATTTCCTACCTGAAAAGGTAAAGAAAAT
IUM-0938   ATTAGTCGGTCTCGAAGCAAACGGAGTGAAGCATGTTATTTAATACGATAATCCGCGTAAAACCTTACCAGAACTTGCATACAAACTATAAAATTTCCTACCTGAAAAGGTAAAGAAAAT
IUM-3986   ATTAGTCGGTCTCGAAGCAAACGGAGTGAAGCATGTTATTTAATACGATAATCCGCGTAAAACCTTACCAGAACTTGCATACAAACTATAAAATTTCCTACCTGAAAAGGTAAAGAAAAT
IUM-4002   ATTAGTCGGTCTCGAAGCAAACGGAGTGAAGCATGTTATTTAATACGATAATCCGCGTAAAACCTTACCAGAACTTGCATACAAACTATAAAATTTCCTACCTGAAAAGGTAAAGAAAAT
IUM-4100   ATTAGTCGGTCTCGAAGCAAACGGAGTGAAGCATGTTATTTAATACGATAATCCGCGTAAAACCTTACCAGAACTTGCATACAAACTATAAAATTTCCTACCTGAAAAGGTAAAGAAAAT
ASI-7117   ATTAGTCGGTCTCGAAGCAAACGGAGTGAAGCATGTTATTTAATACGATAATCCGCGTAAAACCTTACCAGAACTTGCATACAAACTATAAAATTTCCTACCTGAAAAGGTAAAGAAAAT
IUM-4304   ATTAGTCGGTCTCGAAGCAAACGGAGTGAAGCATGTTATTTAATACGATAATCCGCGTAAAACCTTACCAGAACTTGCATACAAACTATAAAATTTCCTACCTGAAAAGGTAAAGAAAAT
IUM-4310   ATTAGTCGGTCTCGAAGCAAACGGAGTGAAGCATGTTATTTAATACGATAATCCGCGTAAAACCTTACCAGAACTTGCATACAAACTATAAAATTTCCTACCTGAAAAGGTAAAGAAAAT
KACC42232  ATTAGTCGGTCTCGAAGCAAACGGAGTGAAGCATGTTATTTAATACGATAATCCGCGTAAGACCTTACCAGAACTTGCATACAAACTATAAAATTTCCTACCTGAAAAGGTAAAGAAAAT
KACC51689  ATTAGTCGGTCTCGAAGCAAACGGAGTGAAGCATGTTATTTAATACGATGATCCGCGTAAAACCTTACCAGAACTTGCATACAAACTATAAAATTTCCTACCTGAAAAGGTAAAGAAAAT
KACC51690  ATTAGTCGGTCTCGAAGCAAACGGAGTGAAGCATGTTATTTAATACGATAATCCGCGTAAAACCTTACCAGAACTTGCATACAAACTATAAAATTTCCTACCTGAAAAGGTAAAGAAAAT
ASI-7037   ATTAGTCGGTCTCGAAGCAAACGGAGTGAAGCATGTTATTTAATACGATAATCCGCGTAAAACCTTACCAGAACTTGCATACAAACTATAAAATTTCCTACCTGAAAAGGTAAAGAAAAT
KCTC 16802 ATTAGTCGGTCTCGAAGCAAACGGAGTGAAGCATGTTATTTAATACGATAATCCGCGTAAAACCTTACCAGAACTTGCATACAAACTATAAAATTTCCTACCTGAAAAGGTAGAGAAAAT
ASI-7068   ATTAGTCGGTCTCGAAGCAAACGGAGTGAAGCATGTTATTTAATACGATAATCCGCGTAAAACCTTACCAGAACTTGCATACAAACTATAAAATTTCCTACCTGAAAGGGTAAAGAAAAT
ASI-7152   ATTAGTCGGTCTCGAAGCAAACGGAGTGAAGCATGTTATTTAATACGATAATCCGCGTAAAACCTTACCAGAACTTGCATACAAACTATAAAATTTCCTACCTGAAAAGGTAAAGAAAAT

                  130       140       150       160       170       180       190       200       210       220       230       240
          ....|....|....|....|....|....|....|....|....|....|....|....|....|....|....|....|....|....|....|....|....|....|....|....|
ASI-7013   ATTAGGAGTGTTTAAAATTTAAATACTTAAGTACAGGTGTTGCATGGCTGTCTTCAGTTCGTGTTGTTAAACATTAGGTTAATTCCACTAACGAACGAAATCCCTGTTATTAATTTATAC
ASI-7074   ATTAGGAGTGTTTAAAATTTAAATACTTAAGTACAGGTGTTGCATGGCTGTCTTCAGTTCGTGTTGTTAAACATTAGGTTAATTCCACTAACGAACGAAATCCCTGTTATTAATTTATAC
ASI-7094   ATTAGGAGTGTTTAAAATTTAAATACTTAAGTACAGGTGTTGCATGGCTGTCTTCAGTTCGTGTTGTTAAACATTAGGTTAATTCCACTAACGAACGAAATCCCTGTTATTAATTTATAC
ASI-7135   ATTAGGAGTGTTTAAAATTTAAATACTTAAGTACAGGTGTTGCATGGCTGTCTTCAGTTCGTGTTGTTAAACATTAGGTTAATTCCACTAACGAACGAAATCCCTGTTATTAATTTATAC
ASI-7146   ATTAGGAGTGTTTAAAATTTAAATACTTAAGTACAGGTGTTGCATGGCTGTCTTCAGTTCGTGTTGTTAAACATTAGGTTAATTCCACTAACGAACGAAATCCCTGTTATTAATTTATAC
ASI-7004   ATTAGGAGTGTTTAAAATTTAAATACTTAAGTACAGGTGTTGCATGGCTGTCTTCAGTTCGTGTTGTTAAACATTAGGTTAATTCCACTAACGAACGAAATCCCTGTTATTAATTTATAC
ASI-7071   ATTAGGAGTGTTTAAAATTTAAATACTTAAGTACAGGTGTTGCATGGCTGTCTTCAGTTCGTGTTGTTAAACATTAGGTTAATTCCACTAACGAACGAAATCCCTGTTATTAATTTATAC
ASI-7091   ATTAGGAGTGTTTAAAATTTAAATACTTAAGTACAGGTGTTGCATGGCTGTCTTCAGTTCGTGTTGTTAAACATTAGGTTAATTCCACTAACGAACGAAATCCCTGTTATTAATTTATAC
IUM-0047   ATTAGGAGTGTTTAAAATTTAAATACTTAAGTACAGGTGTTGCATGGCTGTCTTCAGTTCGTGTTGTTAAACATTAGGTTAATTCCACTAACGAACGAAATCCCTGTTATTAATTTATAC
IUM-0757   ATTAGGAGTGTTTAAAATTTAAATACTTAAGTACAGGTGTTGCATGGCTGTCTTCAGTTCGTGTTGTTAAACATTAGGTTAATTCCACTAACGAACGAAATCCCTGTTATTAATTTATAC
IUM-0938   ATTAGGAGTGTTTAAAATTTAAATACTTAAGTACAGGTGTTGCATGGCTGTCTTCAGTTCGTGTTGTTAAACATTAGGTTAATTCCACTAACGAACGAAATCCCTGTTATTAATTTATAC
IUM-3986   ATTAGGAGTGTTTAAAATTTAAATACTTAAGTACAGGTGTTGCATGGCTGTCTTCAGTTCGTGTTGTTAAACATTAGGTTAATTCCACTAACGAACGAAATCCCTGTTATTAATTTATAC
IUM-4002   ATTAGGAGTGTTTAAAATTTAAATACTTAAGTACAGGTGTTGCATGGCTGTCTTCAGTTCGTGTTGTTAAACATTAGGTTAATTCCACTAACGAACGAAATCCCTGTTATTAATTTATAC
IUM-4100   ATTAGGAGTGTTTAAAATTTAAATACTTAAGTACAGGTGTTGCATGGCTGTCTTCAGTTCGTGTTGTTAAACATTAGGTTAATTCCACTAACGAACGAAATCCCTGTTATTAATTTATAC
ASI-7117   ATTAGGAGTGTTTAAAATTTAAATACTTAAGTACAGGTGTTGCATGGCTGTCTTCAGTTCGTGTTGTTAAACATTAGGTTAATTCCACTAACGAACGAAATCCCTGTTATTAATTTATAC
IUM-4304   ATTAGGAGTGTTTAAAATTTAAATACTTAAGTACAGGTGTTGCATGGCTGTCTTCAGTTCGTGTTGTTAAACATTAGGTTAATTCCACTAACGAACGAAATCCCTGTTATTAATTTATAC
IUM-4310   ATTAGGAGTGTTTAAAATTTAAATACTTAAGTACAGGTGTTGCATGGCTGTCTTCAGTTCGTGTTGTTAAACATTAGGTTAATTCCACTAACGAACGAAATCCCTGTTATTAATTTATAC
KACC42232  ATTAGGAGTGTTTAAAATTTAAATACTTAAGTACAGGTGTTGCATGGCTGTCTTCAGTTCGTGTTGTTAAACATTAGGTTAATTCCACTAACGAACGAAATCCCTGTTATTAATTTATAC
KACC51689  ATTAGGAGTGTTTAAAATTTAAATACTTAAGTACAGGTGTTGCATGGCTGTCTTCAGTTCGTGTTGTTAAACATTAGGTTAATTCCACTAACGAACGAAATCCCTGTTATTAATTTATAC
KACC51690  ATTAGGAGTGTTTAAAATTTAAATACTTAAGTACAGGTGTTGCATGGCTGTCTTCAGTTCGTGTTGTTAAACATTAGGTTAATTCCACTAACGAACGAAATCCCTGTTATTAATTTATAC
ASI-7037   ATTAGGAGTGTTTAAAATTTAAATACTTAAGTACAGGTGTTGCATGGCTGTCTTCAGTTCGTGTTGTTAAACATTAGGTTAATTCCACTAACAAACGAAATCCCTGTTATTAATTTATAC
KCTC 16802 ATTAGGAGTGTTTAAAATTTAAATACTTAAGTACAGGTGTTGCATGGCTGTCTTCAGTTCGTGTTGTTAAACATTAGGTTAATTCCACTAACGAACGAAATCCCTGTTATTAATTTATAC
ASI-7068   ATTAGGAGTGTTTAAAATTTAAATACTTAAGTACAGGTGTTGCATGGCTGTCTTCAGTTCGTGTTGTTAAACATTAGGTTAATTCCACTAACGAACGAAATCCCTGTTATTAATTTATAC
ASI-7152   ATTAGGAGTGTTTAAAATTTAAATACTTAAGTACAGGTGTTGCATGGCTGTCTTCAGTTCGTGTTGTTAAACATTAGGTTAATTCCACTAACGAACGAAATCCCTGTTATTAATTTATAC
```

```
                     250       260       270       280       290       300       310       320       330       340       350       360
            ....|....|....|....|....|....|....|....|....|....|....|....|....|....|....|....|....|....|....|....|....|....|....|....|
ASI-7013    TTAATAACTAATAAATCTGATAGAGATTTAGCGGGGGCTAAGACAAGTCGTTATGGCCCTCATGTTCTGGGCTATAGACGTGCCACAAAAACTTTTACAAAGTGATGCGATACTTCCCAA
ASI-7074    TTAATAACTAATAAATCTGATAGAGATTTAGCGGGGGCTAAGACAAGTCGTTATGGCCCTCATGTTCTGGGCTATAGACGTGCCACAAAAACTTTTACAAAGTGATGCGATACTTCCCAA
ASI-7094    TTAATAACTAATAAATCTGATAGAGATTTAGCGGGGGCTAAGACAAGTCGTTATGGCCCTCATGTTCTGGGCTATAGACGTGCCACAAAAACTTTTACAAAGTGATGCGATACTTCCCAA
ASI-7135    TTAATAACTAATAAATCTGATAGAGATTTAGCGGGGGCTAAGACAAGTCGTTATGGCCCTCATGTTCTGGGCTATAGACGTGCCACAAAAACTTTTACAAAGTGATGCGATACTTCCCAA
ASI-7146    TTAATAACTAATAAATCTGATAGAGATTTAGCGGGGGCTAAGACAAGTCGTTATGGCCCTCATGTTCTGGGCTATAGACGTGCCACAAAAACTTTTACAAAGTGATGCGATACTTCCCAA
ASI-7004    TTAATAACTAATAAATCTGATAGAGATTTAGCGGGGGCTAAGACAAGTCGTTATGGCCCTCATGTTCTGGGCTATAGACGTGCCACAAAAACTTTTACAAAGTGATGCGATACTTCCCAA
ASI-7071    TTAATAACTAATAAATCTGATAGAGATTTAGCGGGGGCTAAGACAAGTCGTTATGGCCCTCATGTTCTGGGCTATAGACGTGCCACAAAAACTTTTACAAAGTGATGCGATACTTCCCAA
ASI-7091    TTAATAACTAATAAATCTGATAGAGATTTAGCGGGGGCTAAGACAAGTCGTTATGGCCCTCATGTTCTGGGCTATAGACGTGCCACAAAAACTTTTACAAAGTGATGCGATACTTCCCAA
IUM-0047    TTAATAACTAATAAATCTGATAGAGATTTAGCGGGGGCTAAGACAAGTCGTTATGGCCCTCATGTTCTGGGCTATAGACGTGCCACAAAAACTTTTACAAAGTGATGCGATACTTCCCAA
IUM-0757    TTAATAACTAATAAATCTGATAGAGATTTAGCGGGGGCTAAGACAAGTCGTTATGGCCCTCATGTTCTGGGCTATAGACGTGCCACAAAAACTTTTACAAAGTGATGCGATACTTCCCAA
IUM-0938    TTAATAACTAATAAATCTGATAGAGATTTAGCGGGGGCTAAGACAAGTCGTTATGGCCCTCATGTTCTGGGCTATAGACGTGCCACAAAAACTTTTACAAAGTGATGCGATACTTCCCAA
IUM-3986    TTAATAACTAATAAATCTGATAGAGATTTAGCGGGGGCTAAGACAAGTCGTTATGGCCCTCATGTTCTGGGCTATAGACGTGCCACAAAAACTTTTACAAAGTGATGCGATACTTCCCAA
IUM-4002    TTAATAACTAATAAATCTGATAGAGATTTAGCGGGGGCTAAGACAAGTCGTTATGGCCCTCATGTTCTGGGCTATAGACGTGCCACAAAAACTTTTACAAAGTGATGCGATACTTCCCAA
IUM-4100    TTAATAACTAATAAATCTGATAGAGATTTAGCGGGGGCTAAGACAAGTCGTTATGGCCCTCATGTTCTGGGCTATAGACGTGCCACAAAAACTTTTACAAAGTGATGCGATACTTCCCAA
ASI-7117    TTAATAACTAATAAATCTGATAGAGATTTAGCGGGGGCTAAGACAAGTCGTTATGGCCCTCATGTTCTGGGCTATAGACGTGCCACAAAAACTTTTACAAAGTGATGCGATACTTCCCAA
IUM-4304    TTAATAACTAATAAATCTGATAGAGATTTAGCGGGGGCTAAGACAAGTCGTTATGGCCCTCATGTTCTGGGCTATAGACGTGCCACAAAAACTTTTACAAAGTGATGCGATACTTCCCAA
IUM-4310    TTAATAACTAATAAATCTGATAGAGATTTAGCGGGGGCTAAGACAAGTCGTTATGGCCCTCATGTTCTGGGCTATAGACGTGCCACAAAAACTTTTACAAAGTGATGCGATACTTCCCAA
KACC42232   TTAATAACTAATAAATCTGATAGAGATTTAGCGGGGGCTAAGACAAGTCGTTATGGCCCTCATGTTCTGGGCTATAGACGTGCCACAAAAACTTTTACAAAGTGATGCGATACTTCCCAA
KACC51689   TTAATAACTAATAAATCTGATAGAGATTTAGCGGGGGCTAAGACAAGTCGTTATGGCCCTCATGTTCTGGGCTATAGACGTGCCACAAAAACTTTTACAAAGTGATGCGATACTTCCCAA
KACC51690   TTAATAACTAATAAATCTGATAGAGATTTAGCGGGGGCTAAGACAAGTCGTTATGGCCCTCATGTTCTGGGCTATAGACGTGCCACAAAAACTTTTACAAAGTGATGCGATACTTCCCAA
ASI-7037    TTAATAACTAATAAATCTGATAGAGATTTAGCGGGGGCTAAGACAAGTCGTTATGGCCCTCATGTTCTGGGCTATAGACGTGCCACAAAAACTTTTACAAAGTGATGCGATACTTCCCAA
KCTC 16802  TTAATAACTAATAAATCTGATAGAGATTTAGCGGGGGCTAAGACAAGTCGTTATGGCCCTCATGTTCTGGGCTATAGACGTGCCACAAAAACTTTTACAAAGTGATGCGATACTTCCCAA
ASI-7068    TTAATAACTAATAAATCTGATAGAGATTTAGCGGGGGCTAAGACAAGTCGTTATGGCCCTCATGTTCTGGGCTATAGACGTGCCACAAAAACTTTTACAAAGTGATGCGATACTTCCCAA
ASI-7152    TTAATAACTAATAAATCTGATAGAGATTTAGCGGGGGCTAAGACAAGTCGTTATGGCCCTCATGTTCTGGGCTATAGACGTGCCACAAAAACTTTTACAAAGTGATGCGATACTTCCCAA

                     370       380       390       400       410       420       430       440       450       460       470       480
            ....|....|....|....|....|....|....|....|....|....|....|....|....|....|....|....|....|....|....|....|....|....|....|....|
ASI-7013    TAGAAGTGGAGCTAATCATTAAAAAAAGTTATTTCTATTAAAAATAAGCCGGATTGTCTCCTGTAATTCGGAGGCATGAAGAAGGAATTCCGAGTCATCGTTGATAAGTAGCGCAACGGT
ASI-7074    TAGAAGTGGAGCTAATCATTAAAAAAAGTTATTTCTATTAAAAATAAGCCGGATTGTCTCCTGTAATTCGGAGGCATGAAGAAGGAATTCCGAGTCATCGTTGATAAGTAGCGCAACGGT
ASI-7094    TAGAAGTGGAGCTAATCATTAAAAAAAGTTATTTCTATTAAAAATAAGCCGGATTGTCTCCTGTAATTCGGAGGCATGAAGAAGGAATTCCGAGTCATCGTTGATAAGTAGCGCAACGGT
ASI-7135    TAGAAGTGGAGCTAATCATTAAAAAAAGTTATTTCTATTAAAAATAAGCCGGATTGTCTCCTGTAATTCGGAGGCATGAAGAAGGAATTCCGAGTCATCGTTGATAAGTAGCGCAACGGT
ASI-7146    TAGAAGTGGAGCTAATCATTAAAAAAAGTTATTTCTATTAAAAATAAGCCGGATTGTCTCCTGTAATTCGGAGGCATGAAGAAGGAATTCCGAGTCATCGTTGATAAGTAGCGCAACGGT
ASI-7004    TAGAAGTGGAGCTAATCATTAAAAAAAGTTATTTCTATTAAAAATAAGCCGGATTGTCTCCTGTAATTCGGAGGCATGAAGAAGGAATTCCGAGTAATCGTTGATAAGTAGCGCAACGGT
ASI-7071    TAGAAGTGGAGCTAATCATTAAAAAAAGTTATTTCTATTAAAAATAAGCCGGATTGTCTCCTGTAATTCGGAGGCATGAAGAAGGAATTCCGAGTAATCGTTGATAAGTAGCGCAACGGT
ASI-7091    TAGAAGTGGAGCTAATCATTAAAAAAAGTTATTTCTATTAAAAATAAGCCGGATTGTCTCCTGTAATTCGGAGGCATGAAGAAGGAATTCCGAGTAATCGTTGATAAGTAGCGCAACGGT
IUM-0047    TAGAAGTGGAGCTAATCATTAAAAAAAGTTATTTATATTAAAAATAAGCCGGATTGTCTCCTGTAATTCGGAGGCATGAAGAAGGAATTCCGAGTAATCGTTGATAAGTAGCGCAACGGT
IUM-0757    TAGAAGTGGAGCTAATCATTAAAAAAAGTTATTTATATTAAAAATAAGCCGGATTGTCTCCTGTAATTCGGAGGCATGAAGAAGGAATTCCGAGTAATCGTTGATAAGTAGCGCAACGGT
IUM-0938    TAGAAGTGGAGCTAATCATTAAAAAAAGTTATTTATATTAAAAATAAGCCGGATTGTCTCCTGTAATTCGGAGGCATGAAGAAGGAATTCCGAGTAATCGTTGATAAGTAGCGCAACGGT
IUM-3986    TAGAAGTGGAGCTAATCATTAAAAAAAGTTATTTATATTAAAAATAAGCCGGATTGTCTCCTGTAATTCGGAGGCATGAAGAAGGAATTCCGAGTAATCGTTGATAAGTAGCGCAACGGT
IUM-4002    TAGAAGTGGAGCTAATCATTAAAAAAAGTTATTTCTATTAAAAATAAGCCGGATTGTCTCCTGTAATTCGGAGGCATGAAGAAGGAATTCCGAGTAATCGTTGATAAGTAGCGCAACGGT
IUM-4100    TAGAAGTGGAGCTAATCATTAAAAAAAGTTATTTCTATTAAAAATAAGCCGGATTGTCTCCTGTAATTCGGAGGCATGAAGAAGGAATTCCGAGTAATCGTTGATAAGTAGCGCAACGGT
ASI-7117    TAGAAGTGGAGCTAATCATTAAAAAAAGTTATTTCTATTAAAAATAAGCCGGATTGTCTCCTGTAATTCGGAGGCATGAAGAAGGAATTCCGAGTAATCGTTGATAAGTAGCGCGACGGT
IUM-4304    TAGAAGTGGAGCTAATCATTAAAAAAAGTTATTTCTATTAAAAATAAGCCGGATTGTCTCCTGTAATTCGGAGGCATGAAGAAGGAATTCCGAGTAATCGTTGATAAGTAGCGCAACGGT
IUM-4310    TAGAAGTGGAGCTAATCATTAAAAAAAGTTATTTCTATTAAAAATAAGCCGGATTGTCTCCTGTAATTCGGAGGCATGAAGAAGGAATTCCGAGTAATCGTTGATAAGTAGCGCAACGGT
KACC42232   TAGAAGTGGAGCTAATCATTAAAAAAAGTTATTTATATTAAAAATAAGCCGGATTGTCTCCTGTAATTCGGAGGCATGAAGAAGGAATTCCGAGTAATCGTTGATAAGTAGCGCAACGGT
KACC51689   TAGAAGTGGAGCTAATCATTAAAGAAAGTTATTTCTATTAAAAATAAGCCGGATTGTCTCCTGTAATTCGGAGGCATGAAGAAGGAATTCCGAGTAATCGTTGATAAGTAGCGCAACGGT
KACC51690   TAGAAGTGGAGCTAATCATTAAAAAAAGTTATTTATATTAAAAATAAGCCGGATTGTCTCCTGTAATTCGGAGGCATGAAGAAGGAATTCCGAGTAATCGTTGATAAGTAGCGCAACGGT
ASI-7037    TAGAAGTGGAGCTAATCATTAAAAAAAGTTATTTCTATTAAAAATAAGCCGGATTGTCTCCTGTAATTCGGAGGCATGAAGAAGGAATTCCGAGTAATCGTTGATAAGTAGCGCAACGGT
KCTC 16802  TAGAAGTGGAGCTAATCATTAAAAAAAGTTATTTCTATTAAAAATAAGCCGGATTGTCTCCTGTAATTCGGAGGCATGAAGAAGGAATTCCGAGTAATCGTTGATAAGTAGCGCAACGGT
ASI-7068    TAGAAGTGGAGCTAATCATTAAAAAAAGTTATTTATATTAAAAATAAGCCGGATTGTCTCCTGTAATTCGGAGGCATGAAGAAGGAATTCCGAGTAATCGTTGATAAGTAGCGCAACGGT
ASI-7152    TAGAAGTGGAGCTAATCATTAAAAAAAGTTATTTCTATTAAAAATAAGCCGGATTGTCTCCTGTAATTCGGAGGCATGAAGAAGGAATTCCGAGTAATCGTTGATAAGTAGCGCAACGGT
```

```
                   490       500       510       520       530       540       550       560       570       580       590       600
ASI-7013    GAAGCTAGTTTCGTGATGAACTAACTGTCTGTCGCTGGGAAGAAGCTTTTAGTGGAGGAAACTGGAGTAAGATTATGTTTTTTCATAGTAACTCATTGCTTTAAGTAAAGTATTTTTAGT
ASI-7074    GAAGCTAGTTTCGTGATGAACTAACTGTCTGTCGCTGGGAAGAAGCTTTTAGTGGAGGAAACTGGAGTAAGATTATGTTTTTTCATAGTAACTCATTGCTTTAAGTAAAGTATTTTTAGT
ASI-7094    GAAGCTAGTTTCGTGATGAACTAACTGTCTGTCGCTGGGAAGAAGCTTTTAGTGGAGGAAACTGGAGTAAGATTATGTTTTTTCATAGTAACTCATTGCTTTAAGTAAAGTATTTTTAGT
ASI-7135    GAAGCTAGTTTCGTGATGAACTAACTGTCTGTCGCTGGGAAGAAGCTTTTAGTGGAGGAAACTGGAGTAAGATTATGTTTTTTCATAGTAACTCATTGCTTTAAGTAAAGTATTTTTAGT
ASI-7146    GAAGCTAGTTTCGTGATGAACTAACTGTCTGTCGCTGGGAAGAAGCTTTTAGTGGAGGAAACTGGAGTAAGATTATGTTTTTTCATAGTAACTCATTGCTTTAAGTAAAGTATTTTTAGT
ASI-7004    GAAGCTAGTTTCGTGATGAACTAACTGTCTGTCGCTGGGAAGAAGCTTTTAGTGGAGGAAACTGGAGTAAGATTATGTTTTTTCATAGTAACTCATTGCTTTAAGTAAAGTATTTTTAGT
ASI-7071    GAAGCTAGTTTCGTGATGAACTAACTGTCTGTCGCTGGGAAGAAGCTTTTAGTGGAGGAAACTGGAGTAAGATTATGTTTTTTCATAGTAACTCATTGCTTTAAGTAAAGTATTTTTAGT
ASI-7091    GAAGCTAGTTTCGTGATGAACTAACTGTCTGTCGCTGGGAAGAAGCTTTTAGTGGAGGAAACTGGAGTAAGATTATGTTTTTTCATAGTAACTCATTGCTTTAAGTAAAGTATTTTTAGT
IUM-0047    GAAGCTAGTTTCGTGATGAACTAACTGTCTGTCGCTGGGAAGAAGCTTTTAGTGGAGGAAACTGGAGTAAGATTATGTTTTTTCATAGTAACTCATTGCTTTAAGTAAAGTATTTTTAGT
IUM-0757    GAAGCTAGTTTCGTGATGAACTAACTGTCTGTCGCTGGGAAGAAGCTTTTAGTGGAGGAAACTGGAGTAAGATTATGTTTTTTCATAGTAACTCATTGCTTTAAGTAAAGTATTTTTAGT
IUM-0938    GAAGCTAGTTTCGTGATGAACTAACTGTCTGTCGCTGGGAAGAAGCTTTTAGTGGAGGAAACTGGAGTAAGATTATGTTTTTTCATAGTAACTCATTGCTTTAAGTAAAGTATTTTTAGT
IUM-3986    GAAGCTAGTTTCGTGATGAACTAACTGTCTGTCGCTGGGAAGAAGCTTTTAGTGGAGGAAACTGGAGTAAGATTATGTTTTTTCATAGTAACTCATTGCTTTAAGTAAAGTATTTTTAGT
IUM-4002    GAAGCTAGTTTCGTGATGAACTAACTGTCTGTCGCTGGGAAGAAGCTTTTAGTGGAGGAAACTGGAGTAAGATTATGTTTTTTCATAGTAACTCATTGCTTTAAGTAAAGTATTTTTAGT
IUM-4100    GAAGCTAGTTTCGTGATGAACTAACTGTCTGTCGCTGGGAAGAAGCTTTTAGTGGAGGAAACTGGAGTAAGATTATGTTTTTTCATAGTAACTCATTGCTTTAAGTAAAGTATTTTTAGT
ASI-7117    GAAGCTAGTTTCGTGATGAACTAACTGTCTGTCGCTGGGAAGAAGCTTTTAGTGGAGGAAACTGGAGTAAGATTATGTTTTTTCATAGTAACTCATTGCTTTAAGTAAAGTATTTTTAGT
IUM-4304    GAAGCTAGTTTCGTGATGAACTAACTGTCTGTCGCTGGGAAGAAGCTTTTAGTGGAGGAAACTGGAGTAAGATTATGTTTTTTCATAGTAACTCATTGCTTTAAGTAAAGTATTTTTAGT
IUM-4310    GAAGCTAGTTTCGTGATGAACTAACTGTCTGTCGCTGGGAAGAAGCTTTTAGTGGAGGAAACTGGAGTAAGATTATGTTTTTTCATAGTAACTCATTGCTTTAAGTAAAGTATTTTTAGT
KACC42232   GAAGCTAGTTTCGTGATGAACTAACTGTCTGTCGCTGGGAAGAAGCTTTTAGTGGAGGAAACTGGAGTAAGATTATGTTTTTTCATAGTAACTCATTGCTTTAAGTAAAGTATTTTTAGT
KACC51689   GAAGCTAGTTTCGTGATGAACTAACTGTCTGTCGCTGGGAAGAAGCTTTTAGTGGAGGAAACTGGAGTAAGATTATGTTTTTTCATAGTAACTCATTGCTTTAAGTAAAGTATTTTTAGT
KACC51690   GAAGCTAGTTTCGTGATGAACTAACTGTCTGTCGCTGGGAAGAAGCTTTTAGTGGAGGAAACTGGAGTAAGATTATGTTTTTTCATAGTAACTCATTGCTTTAAGTAAAGTATTTTTAGT
ASI-7037    GAAGCTAGTTTCGTGATGAACTAACTGTCTGTCGCTGGGAAGAAGCTTTTAGTGGAGGAAACTGGAGTAAGATTATGTTTTTTCATAGTAACTCATTGCTTTAAGTAAAGTATTTTTAGT
KCTC 16802  GAAGCTAGTTTCGTGATGAACTAACTGTCTGTCGCTGGGAAGAAGCTTTTAGTGGAGGAAACTGGAGTAAGATTATGTTTTTTCATAGTAACTCATTGCTTTAAGTAAAGTATTTTTAGT
ASI-7068    GAAGCTAGTTTCGTGATGAACTAACTGTCTGTCGCTGGGAAGAAGCTTTTAGTGGAGGAAACTGGAGTAAGATTATGTTTTTTCATAGTAACTCATTGCTTTAAGTAAAGTATTTTTAGT
ASI-7152    GAAGCTAGTTTCGTGATGAACTAACTGTCTGTCGCTGGGAAGAAGCTTTTAGTGGAGGAAACTGGAGTAAGATTATGTTTTTTCATAGTAACTCATTGCTTTAAGTAAAGTATTTTTAGT

                 610       620       630       640       650       660
ASI-7013    ATTATGATAAACTTATTGGATTTAGTGAATTCATTAAGGTAACATCTCAAAAGTCATAGCA
ASI-7074    ATTATGATAAACTTATTGGATTTAGTGAATTCATTAAGGTAACATCTCAAAAGTCATAGCA
ASI-7094    ATTATGATAAACTTATTGGATTTAGTGAATTCATTAAGGTAACATCTCAAAAGTCATAGCA
ASI-7135    ATTATGATAAACTTATTGGATTTAGTGAATTCATTAAGGTAACATCTCAAAAGTCATAGCA
ASI-7146    ATTATGATAAACTTATTGGATTTAGTGAATTCATTAAGGTAACATCTCAAAAGTCATAGCA
ASI-7004    ATTATGATAAACTTATTGGATTTAGTGAATTCATTAAGGTAACATCTCAAAAGTCATAGCA
ASI-7071    ATTATGATAAACTTATTGGATTTAGTGAATTCATTAAGGTAACATCTCAAAAGTCATAGCA
ASI-7091    ATTATGATAAACTTATTGGATTTAGTGAATTCATTAAGGTAACATCTCAAAAGTCATAGCA
IUM-0047    ATTATGATAAACTTATTGGATTTAGTGAATTCATTAAGGTAACATCTCAAAAGTCATAGCA
IUM-0757    ATTATGATAAACTTATTGGATTTAGTGAATTCATTAAGGTAACATCTCAAAAGTCATAGCA
IUM-0938    ATTATGATAAACTTATTGGATTTAGTGAATTCATTAAGGTAACATCTCAAAAGTCATAGCA
IUM-3986    ATTATGATAAACTTATTGGATTTAGTGAATTCATTAAGGTAACATCTCAAAAGTCATAGCA
IUM-4002    ATTATGATAAACTTATTGGATTTAGTGAATTCATTAAGGTAACATCTCAAAAGTCATAGCA
IUM-4100    ATTATGATAAACTTATTGGATTTAGTGAATTCATTAAGGTAACATCTCAAAAGTCATAGCA
ASI-7117    ATTATGATAAACTTATTGGATTTAGTGAATTCATTAAGGTAACATCTCAAAAGTCATAGCA
IUM-4304    ATTATGATAAACTTATTGGATTTAGTGAATTCATTAAGGTAACATCTCAAAAGTCATAGCA
IUM-4310    ATTATGATAAACTTATTGGATTTAGTGAATTCATTAAGGTAACATCTCAAAAGTCATAGCA
KACC42232   ATTATGATAAACTTATTGGATTTAGTGAATTCATTAAGGTAACATCTCAAAAGTCATAGCA
KACC51689   ATTATGATAAACTTATTGGATTTAGTGAATTCATTAAGGTAACATCTCAAAAGTCATAGCA
KACC51690   ATTATGATAAACTTATTGGATTTAGTGAATTCATTAAGGTAACATCTCAAAAGTCATAGCA
ASI-7037    ATTATGATAAACTTATTGGATTTAGTGAATTCATTAAGGTAACATCTCAAAAGTCATAGCA
KCTC 16802  ATTATGATAAACTTATTGGATTTAGTGAATTCATTAAGGTAACATCTCAAAAGTCATAGCA
ASI-7068    ATTATGATAAACTTATTGGATTTAGTGAATTCATTAAGGTAACATCTCAAAAGTCATAGCA
ASI-7152    ATTATGATAAACTTATTGGATTTAGTGAATTCATTAAGGTAACATCTCAAAAGTCATAGCA
```

Figure S1. Alignment of partial mitochondrial SSU rDNA sequences of *Ganoderma lucidum* strains. Red arrow indicates the SNP position found from antler-shaped *G. lucidum* strains.
